# Analysis of Circulating Tumor DNA in Synchronous Metastatic Colorectal Cancer at Diagnosis Predicts Overall Patient Survival

**DOI:** 10.3390/ijms24098438

**Published:** 2023-05-08

**Authors:** José María Sayagués, Juan Carlos Montero, Andrea Jiménez-Pérez, Sofía del Carmen, Marta Rodríguez, Rosario Vidal Tocino, Enrique Montero, Julia Sanz, Mar Abad

**Affiliations:** 1 Department of Pathology and IBSAL, University Hospital of Salamanca, University of Salamanca, 37007 Salamanca, Spain; jcmon@usal.es (J.C.M.); abjimenez@saludcastillayleon.es (A.J.-P.); martarodriguez@saludcastillayleon.es (M.R.); 2 Biomedical Research Networking Centers-Oncology (CIBERONC), 28029 Madrid, Spain; 3 Department of Pathology, University Hospital of Marqués de Valdecilla, 39008 Santander, Spain; sofia.delcarmen@scsalud.es; 4 Department of Oncology and IBSAL, University Hospital of Salamanca, 37007 Salamanca, Spain; mrvidal@saludcastillayleon.es; 5 Department of Pathology, University Hospital of Zamora, 49071 Zamora, Spain; emonteroma@saludcastillayleon.es; 6 Department of Pathology, Puerto Real University Hospital, 11510 Cadiz, Spain; jsrepetto@saludcastillayleon.es

**Keywords:** synchronous metastatic colorectal cancer, liquid biopsy, *KRAS*, *NRAS*, *PIK3CA*, *BRAF*, anti-EGFR, anti-VEGF

## Abstract

Sporadic colorectal cancer (sCRC) initially presents as metastatic tumors in 25–30% of patients. The 5-year overall survival (OS) in patients with metastatic sCRC is 50%, falling to 10% in patients presenting with synchronous metastatic disease (stage IV). In this study, we systematically analyzed the mutations of *RAS*, *PIK3CA* and *BRAF* genes in circulating tumor DNA (ctDNA) and tumoral tissue DNA (ttDNA) from 51 synchronous metastatic colorectal carcinoma (SMCC) patients by real-time PCR, and their relationship with the clinical, biological and histological features of disease at diagnosis. The highest frequency of mutations detected was in the *KRAS* gene, in tumor biopsies and plasma samples, followed by mutations of the *PIK3CA*, *NRAS* and *BRAF* genes. Overall, plasma systematically contained those genetic abnormalities observed in the tumor biopsy sample from the same subject, the largest discrepancies detected between the tumor biopsy and plasma from the same patient being for mutations in the *KRAS* and *PIK3CA* genes, with concordances of genotyping results between ttDNA and ctDNA at diagnosis of 75% and 84%, respectively. Of the 51 SMCC patients in the study, 25 (49%) showed mutations in at least 1 of the 4 genes analyzed in patient plasma. From the prognostic point of view, the presence and number of the most common mutations in the *RAS, PIK3CA* and *BRAF* genes in plasma from SMCC patients are independent prognostic factors for OS. Determination of the mutational status of ctDNA in SMCC could be a key tool for the clinical management of patients.

## 1. Introduction

Approximately 25–30% of patients with sporadic colorectal cancer (sCRC) initially present as metastatic tumors (stage IV). Of the other patients, who are mainly diagnosed in stages II or III, 40% will progress to more advanced stages and metastatic processes, the liver being the most common site for metastatic spread of the primary tumor [1]. Overall, the 5-year overall survival (OS) in patients with metastatic CRC is 50%, falling to 10% in stage IV patients (synchronous metastatic disease) [1].

It is well known that the pathogenesis of sCRC is due to the sequential appearance of abnormalities at the genetic level that, from a preneoplastic stage, lead to a generalized alteration of the genome by the clonal expansion of cells carrying mutations that frequently affect *APC*, *RAS*, *TP53* and/or *DCC* genes [2]. However, genetic alterations participating in the metastatic process, through which tumor cells of the primary tumor can colonize other tissues, remain to be identified. Recent genetic studies of metastatic tumors carried out by our research group suggest that the metastatic potential resides in the primary tumor itself [3]. Thus, we and other researchers have recently shown specific genomic alterations that are already present in the primary tumor of metastatic sCRC patients (e.g., del(17p) and del(22q)), as well as the differential expression of 28 genes (e.g., dysregulated transcripts of *ADH1B*, *BST2* and *FER1L4* genes) between metastatic and non-metastatic sCRC tumors [4]. 

Various therapeutic protocols based on general chemotherapeutic agents have been applied for the treatment of metastatic sCRC, and, more recently, new protocols have been implemented that consist of the combined use of chemotherapy with monoclonal antibodies against specific oncogenic targets. Although at the beginning of the 1980s the median survival of patients with disseminated disease was 6 months, this was extended to 1 year with the appearance of regimens based on 5-fluorouracil. Subsequently, this was extended to as much as 20 months by the addition of irinotecan and oxaliplatin. More recently, and as a result of oncology entering the molecular era, the following monoclonal antibodies have been included in clinical practice: bevacizumab, anti-vascular endothelial growth factor (VEGF), and cetuximab and panitumumab, two anti-epithelial growth factor receptors (EGFRs) that, combined with cytostatic and surgical treatment, extend patient survival to approximately 24 months. However, these two anti-EGFRs are only active when the tumor cells of the patient do not present mutations at the level of the *RAS*, *PIK3CA* and *BRAF* genes. Therefore, triple-negative patients (*KRAS*, *BRAF* and *PI3K3CA*; “wild type”) are those who would benefit the most from these therapies [5].

Currently, mutations of these genes are evaluated in DNA from formalin-fixed, paraffin-embedded tumor tissue (tumor resection or biopsies). However, it is not always possible to extract sufficient DNA of adequate quality for mutational studies [6]. DNA fragmentation is very frequent in paraffin samples, which can affect the integrity of the molecule. In recent years, the analysis of circulating tumor DNA (ctDNA) has opened up a new avenue for the study and monitoring of patient tumor burden. ctDNA can be isolated from plasma or serum and has the potential to be a viable starting material for identifying genetic markers for disease diagnosis and recurrence. In addition, ctDNA analysis provides a real-time assessment of the mutational status (presence of pathogenic mutations) of genes involved in disease pathogenesis. On the other hand, ctDNA mutational analysis may also provide a better representation of the tumor because it has the potential to generate information about all subclones of a tumor, which may contain DNA fragments from the primary tumor and the distant metastatic tumors [7]. 

In the present study, we investigated the prognostic value of the mutational status of the *KRAS*, *NRAS*, *PIK3CA* and *BRAF* genes detected in tumor biopsies and plasma samples from 51 synchronous metastatic colorectal cancer (SMCC) patients. Overall, our results show that *KRAS* mutations, determined both in tumor tissue and in patient plasma, showed a significant adverse influence on OS in univariate analysis. However, in the multivariate analysis, only the mutations identified in the plasma of the SMCC patients maintained statistical significance, behaving as an independent prognostic factor for OS. 

## 2. Results 

### 2.1. Frequency, Type and Concordance of KRAS, NRAS, PIK3CA and BRAF Mutations Detected in Tumor Tissue and Plasma of 51 Patients with Synchronous Metastatic Colorectal Cancer (SMCC)

**Figure 1:**
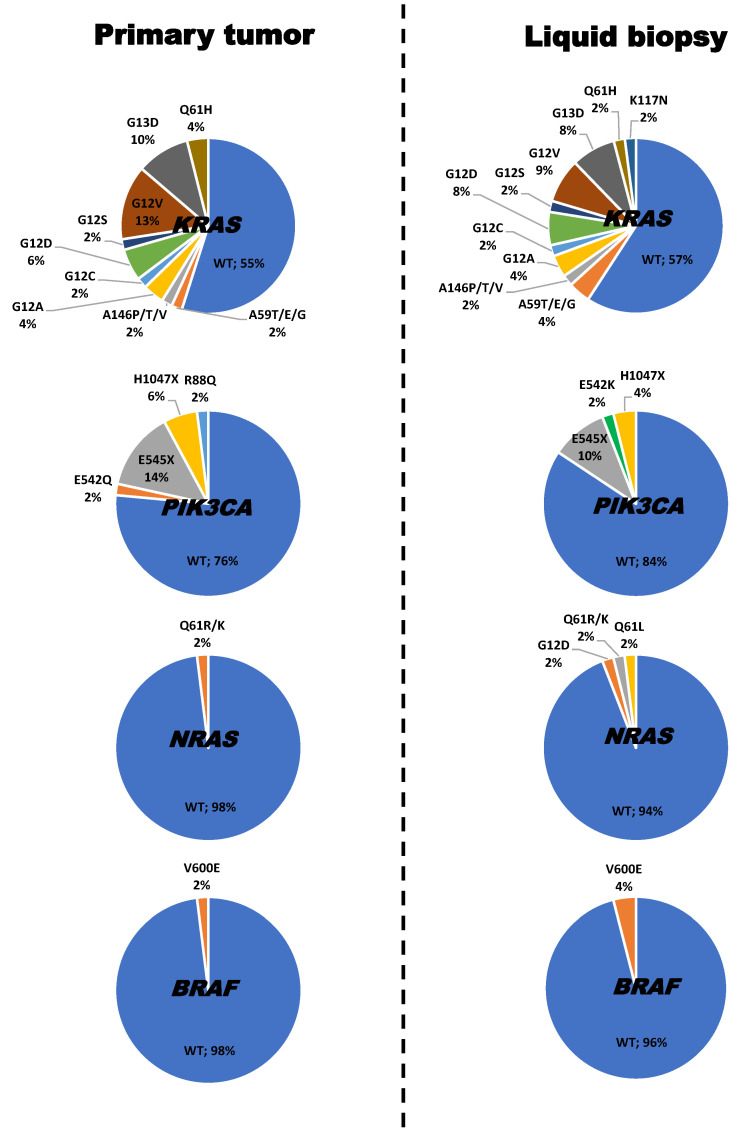
Frequency and type of mutations detected in the *KRAS*, *NRAS*, *PIK3CA* and *BRAF* genes in paired primary tumor and plasma (liquid biopsy) samples from 51 patients with synchronous metastatic colorectal cancer (SMCC) at diagnosis.

The highest frequency of mutations occurred in the *KRAS* gene, in the tumor biopsy (45% of cases) and plasma (43% of patients), the G12V and G13D mutations being the most frequently detected (Figure 1), followed by mutations of the *PIK3CA* (24% in tumor biopsy and 16% in plasma), *NRAS* (2% and 6%) and *BRAF* genes (2% and 4%), and the ES45X, Q61R/K and V600E mutations (Figure 1).

We also observed a statistically significant correlation among the mutational status of the *KRAS*, *NRAS*, *PIK3CA* and *BRAF* genes detected in plasma and tumoral tissue at diagnosis of the disease (Table 1). Overall, plasma systematically contained those genetic abnormalities observed in the tumor biopsy sample from the same subject. However, tumor biopsies from many cases (up to 5 out of 51) showed *KRAS* mutations that were not found in their corresponding plasma, while the plasma from 4 cases displayed *KRAS* mutations that were not detected in their corresponding tumoral biopsy sample (Figure 1 and Supplementary Table S1). Two patients showed *NRAS* mutations in the plasma, but not in their tumor biopsy sample, while only one patient showed such a discrepancy for *BRAF* gene mutations (Table 1). The concordances of genotyping results between tDNA and ctDNA at diagnosis were 75%, 96%, 98% and 84% for the *KRAS*, *NRAS*, *BRAF* and *PIK3CA* genes, respectively. 

### 2.2. Association between Mutational Status Detected in Patient Plasma and Other Features of the Disease

Of the 51 SMCC patients included in the present study, 26 (51%) displayed mutations for at least 1 of the 4 genes analyzed in the plasma (positive biopsy). Overall, most patients with a positive biopsy had a tumor in the right colon or rectum with abnormally high CEA serum levels (>7.5 ng/mL; *p* = 0.002), and they showed a higher frequency of deaths (*p* = 0.009) in association with significantly shortened patient overall survival (OS) (median of 24 months; *p* ≤ 0.001). As expected, a significant correlation was detected between the mutations detected in plasma and those identified in the primary tumor. In contrast, no significant differences were found between positive and negative biopsy SMCC patients, having taken gender and patient age into account (Table 1).

### 2.3. Impact of Liquid Biopsy on Patient OS

**Figure 2:**
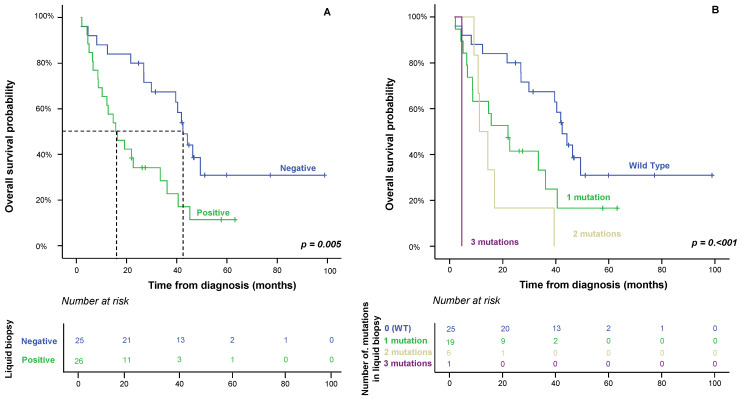
Identification of at least one mutation in the *KRAS*, *NRAS*, *PIK3CA* and/or *BRAF* genes in the plasma of synchronous metastatic colorectal cancer patients (positive liquid biopsy) at diagnosis (Panel **A**), and the increase in the number of mutations detected (Panel **B**), show a significant impact on overall survival in the univariate (*p* ≤ 0.005) and multivariate (*p* ≤ 0.007) analyses.

From the prognostic point of view, the clinical, biological and pathological characteristics of the disease that displayed a significant adverse influence on OS in the univariate analysis included increased (>7.5 ng/mL) CEA serum levels (*p* = 0.073), *KRAS* mutations determined in both primary tumor and plasma (*p* = 0.05 and *p* = 0.004, respectively), positive liquid biopsy at diagnosis (*p* = 0.05) and, interestingly, the number of mutations detected in plasma at diagnosis (*p* ≤ 0.001) (Table 2), the determination of the *KRAS* mutation in plasma being a good prognostic factor in the univariate analysis. However, multivariate analysis of the prognostic factors for OS showed that the presence and number of mutations detected in the plasma at diagnosis were the only independent variables that predicted an adverse outcome (Table 1 and Figure 2).

## 3. Discussion

The chances of a cure for patients with sporadic colorectal cancer (sCRC) who develop distant metastases to the liver and other organs at the time of diagnosis are dramatically low. Even though we now have a much better understanding of the genetic mechanisms that control the early stages of disease, the factors involved in metastatic processes remain poorly understood. In this context, it is of utmost importance to develop methods capable of identifying patients at high risk for an adverse outcome or of predicting the onset of metastasis, the main cause of death of sCRC patients. In recent years, various studies have demonstrated the prognostic value of cfDNA in sCRC, raising the possibility that their analysis might identify patients with localized tumors who are at risk of recurrence [8]. Other studies have shown that molecular analysis of the tumor through liquid biopsies provides information about the changes in the RAS mutational status due to tumoral heterogeneity and selective pressure by targeted therapies throughout the course of the disease [9,10]. However, the significance of liquid biopsy in the clinical management of patients with synchronous metastases remains to be elucidated.

In this study, we describe the frequency and type of mutations found in biopsies of tumor tissue and in peripheral blood at the time of diagnosis of the disease to determine the most appropriate therapy for each synchronous metastatic colorectal cancer (SMCC) patient: chemotherapy alone or combined with monoclonal antibodies, anti-epidermal growth factor receptor (EGFR) or anti-vascular endothelial growth factor receptor (VEGFR). In turn, we show how the presence of mutations and their accumulation influence the overall survival (OS) of the patient, regardless of the medical treatment received. To the best of our knowledge, this is the first report demonstrating that the co-occurrence of mutations in genes involved in the EGFR signaling pathway in peripheral blood from SMCC patients is associated with a significantly short OS. Consistent with our observations, Kawazoe et al. [11], in a retrospective observational study, described the mutational status of *KRAS*, *NRAS*, *BRAF* or *PIK3CA* in tissue biopsies from 264 patients with mCRC, demonstrating that patients with any mutation in these genes had a shorter survival outcome after receiving treatments with monoclonal antibodies. However, the series of patients studied was not homogeneous since it included patients with synchronous and metachronous metastases. In addition, they did not determine the mutational status of the genes using liquid biopsy techniques, which could resolve the possible genetic heterogeneity present in this type of tumor and find other genetic lesions present in the metastatic samples [12]. In this regard, several studies that have explored ctDNA levels in mCRC have indicated that elevated ctDNA levels are correlated with poorer survival. Thus, Yang et al. [7] analyzed ctDNA levels in 47 CRC patients in early or late cancer stages and found that stage IV patients had significantly higher ctDNA concentrations than stage I patients. Similarly, Güttlein’s work [13] also supports the utility of *KRAS*, *NRAS* and *BRAF* analysis in liquid biopsy from CRC patients with synchronous and metachronous metastases, finding an association between *RAS/BRAF*-mutated patients and a shorter OS. These studies support the implication that ctDNA characteristics could help in the clinical management of metastatic patients. 

In the present study, all patients in our cohort had both tissue and plasma available at diagnosis. The concordance between tissue and plasma was approximately 75% when the *KRAS* gene was analyzed, as previously observed. In an earlier study, Rodon et al. [12] found a 76.5% concordance of genotyping results using NGS in 18 tissue and blood samples of patients with locally advanced CRC or mCRC. Similarly, Erve et al. [14] observed a 93% concordance between tumoral tissue DNA (ttDNA) and liquid biopsy *RAS/BRAF* ctDNA in their analysis of 100 sCRC patients with liver metastases. Kagawa et al. [15] investigated the concordance of the RAS status between Digital PCR (OncoBEAM™) and tissue biopsies in 221 mCRC patients, and found concordance ratios between 64% and 91%, depending on whether the metastatic site was the liver, peritoneum or lung. Discrepant results could be explained by the acquisition of the mutation during the disease’s progression to the liver. However, *KRAS* mutation occurs in early stages of carcinogenesis [16], although it is not uncommon for the *KRAS* mutation to be acquired after metastasis. In our series, 4 (8%) patients had a *KRAS* mutation that was detected in liquid biopsy, but not in the tumoral biopsy. Taniguchi et al. [17] demonstrated that metastatic lesions harbored diverse acquired mutations of *KRAS* in primary tumors. 

The results of our study show that the best method for predicting the disease prognosis is the mutational characterization of the EGFR signaling pathway genes in the patient’s plasma (vs. tumor tissue). Testing ctDNA in peripheral blood (liquid biopsy) has emerged as a new and useful tool in the diagnosis and follow-up of sCRC patients. Detection of the mutational status of genes involved in the EGFR signaling pathway genes in ctDNA from blood samples seems to be a simple and non-invasive alternative to testing primary tumors. In addition, it is an easy and inexpensive technique to perform in clinical laboratories, and it can be carried out easily at different times during the course of the disease, providing information about dynamic changes in the genotype of mCRC cells [18], successfully resolving the possible spatial and temporal heterogeneity present in this type of tumor [19]. The absolute amount of ctDNA depends on the stage [20] and location [17] of the tumor. Surgery and medical treatment both modify ctDNA levels, which act as a surrogate biomarker of the response to anti-EGFR treatments and progression-free survival (PFS) in sCRC patients with localized disease. However, the clinical significance and impact of anti-angiogenics (anti-VEGFR agents) [21] in synchronous metastatic disease remain to be elucidated. Most studies have focused on the progression of the primary tumor after its surgical resection. In the metastatic process of sCRC, tumor hypoxia produces overexpression of the hypoxia-inducible factor-1 (HIF-1) and release of the VEGF, which stimulates neo-angiogenesis to ensure the survival of the tumor cells [22]. In addition, VEGF expression under hypoxic conditions can also be stimulated by the EGFR signaling pathway, which, in turn, is frequently activated by the appearance of mutations in the *KRAS*, *NRAS*, *BRAF* or *PIK3CA* genes [23]. Currently, the decision to administer a treatment is based solely on the molecular information obtained from the initial tumor biopsy. However, published results show that metastatic disease can undergo mutational variations that can condition the choice of treatment by the selection of resistant clones after administering systemic therapy [19,24,25]. In fact, the study carried out by Erik et al. suggests that analysis of plasma-derived ctDNA in patients with mCRC may identify additional RAS mutations that would improve patient selection for anti-EGFR therapies [25]. Klein et al. [9] demonstrated that although systemic therapies kill many tumor cells, resistant tumor cell clones are selected, avoiding the cytotoxic action of the administered therapy. It has been hypothesized that the tumor hypoxia produced in the metastatic process could also contribute to this selection of clones capable of surviving in conditions of low oxygen supply. Half of the patients analyzed by Gazzaniga et al. [26] changed their mutational status to become RAS wild-type after receiving antiangiogenic therapy. Garcia et al. [27] detected a RAS mutational change in 74% of patients after a median of 3 months of bevacizumab treatment. Even traditional chemotherapy has been linked to the modification of the RAS mutational status. Therefore, the study of molecular profiling using liquid biopsy could be a key tool for predicting OS in patients with SMCC treated with anti-EGFR and anti-VEGFR drugs. It should be noted that several studies of genome sequencing have observed that one in five healthy individuals may carry disease-related genetic mutations [16,28]. However, none of the mutations detected in these studies were present in the genes studied here.

Limited information is available on the feasibility and clinical potential of ctDNA analysis in non-metastatic CRC cancer and/or in early stage of the disease. The results of these studies are difficult to interpret and compare, especially due to significant heterogeneity regarding the patient’s series analyzed. While Tie et al. [29] reported that ctDNA significantly outperforms standard clinicopathologic characteristics as a prognostic marker in stage II patients, Sclafani et al. [30] failed to predict the prognosis with the detection of *KRAS* mutation in ctDNA of locally advanced rectal cancer patients. Larger series are needed to better address the role of ctDNA as a prognostic or predictive tool in this regard. 

In summary, our results show that the presence of mutations in the *RAS*, *PIK3CA* and/or *BRAF* genes in the plasma of patients with SMCC is an independent prognostic factor for OS. The data also provide evidence that the number of mutations is negatively correlated with the OS of patients, regardless of the treatment they receive. Molecular information obtained by ctDNA analysis could be useful in our daily clinical practice to improve prognostic assessments and to guide clinical decision making in SMCC patients. Additional prospective studies are required in larger series to confirm the utility of the proposed predictive model.

## 4. Materials and Methods

### 4.1. Patients and Samples

Peripheral blood and endoscopically acquired tumor tissue biopsies from the primary tumor were obtained from 51 consecutive patients with synchronous metastatic colorectal cancer (SMCC) between June 2014 and September 2017 in the Department of Surgery of the University Hospital of Salamanca (Salamanca, Spain), before administering any cytotoxic therapy and after each subject had given their informed consent, in accordance with the Declaration of Helsinki. In total, 37 were men and 14 women, with a median age 66 years (range 43–83 years). Diagnosis and classification of the tumor were made according to the AJCC criteria [31]. All cases were adenocarcinomas. Median follow-up at the close of the study was 40 months (range 30–50 months). Patient clinical, laboratory and follow-up data are summarized in Table 3. 

The study was approved by the local ethics committee of the University Hospital of Salamanca (Salamanca, Spain).

### 4.2. Tissue-Based RAS, BRAF and PIK3CA Mutation Analysis

Tumor tissue biopsies were collected and mutations were sought at diagnosis. Hematoxylin-eosin (HE)-stained slides were reviewed. The DNA was isolated from a paraffin block containing at least 70% of tumor cells. One or two 10 μm-thick formalin-fixed paraffin-embedded (FFPE) tumor tissue sections were deparaffinized with xylene for 5 min at room temperature (RT), dehydrated in absolute alcohol for 5 min at RT and allowed to air-dry completely for 10 min. Later, DNA was isolated using the Cobas DNA Sample Preparation Kit (Roche, Branchburg, NJ, USA) following the manufacturer’s instructions.

### 4.3. Blood-Based RAS, BRAF and PIK3CA Mutation Analysis 

In parallel, cfDNA was isolated from 2 mL of plasma obtained from each patient (*n* = 51) at diagnosis, using a Cobas^®^ cfDNA Sample Preparation Kit (Roche, Branchburg, NJ, USA) and following the manufacturer’s instructions. 

A Nanodrop UV spectrophotometer (Thermo Fisher Scientific, Wilmington, DE, USA) was used to verify the quality and quantity of the extracted DNA from both tumor tissue and patient plasma. Amplification and detection were performed with an Automated Cobas z480 analyzer instrument (Roche Molecular System Inc., Pleasanton, CA, USA). The real-time PCR tests examined the most common mutations in codons 12, 13, 59, 61, 117 and 146 in the *KRAS* and *NRAS* genes; the V600E *BRAF* mutation; and in exons 2, 5, 8, 10 and 21 of *PIK3CA* mutations. The tests to detect these mutations both in tumor tissue and in the patient’s peripheral blood were purchased from Roche (Branchburg, NJ, USA): the KRAS v2 mutation test (LSR), BRAF/NRAS mutation test (LSR) and the Cobas mutation test PIK3CA. Data were analyzed according to the manufacturer’s instructions by uploading the .ixo files to the online LSR Data Analysis tool: https://lifescience.roche.com/en_nl/brands/oncology-research-kits.html (accession on 18 January 2023).

### 4.4. Statistical Analyses

The mean, standard deviation (SD) and range of all continuous variables were calculated, and dichotomous variables were reported as frequencies and percentages. To evaluate the statistical significance of group differences, Student’s *t*-test and the Mann–Whitney U test were used for normally and non-normally distributed continuous variables, respectively. For dichotomous variables, the X^2^ test was used. Overall survival (OS) curves were plotted according to the Kaplan–Meier method, and the log-rank test (one-sided) was used to determine the statistical significance of the differences between survival curves. Multivariate analysis of prognostic factors for OS were identified by multivariate stepwise Cox regression, using forward selection and considering only those variables that showed a significant association with OS in the univariate analysis. All analyses were carried out using IBM SPSS Statistics v.22 (IBM Corp., Armonk, NY, USA). Statistical significance was concluded for values of *p* (Pearson-corrected where appropriate) of <0.05.

## Figures and Tables

**Table 1:** Clinical, biological and genetic characteristics of synchronous metastatic colorectal cancer (SMCC) patients with positive (*n* = 26) and negative (*n* = 25) liquid biopsy at diagnosis. Patients with a positive liquid biopsy were considered when at least one mutation in the *KRAS*, *NRAS*, *PIK3CA* and/or *BRAF* genes was detected in the patient’s plasma.

Variable	Negative Liquid Biopsy Patients (*n* = 25)	Positive Liquid Biopsy Patients (*n* = 26)	*p*	Total (*n* = 51)
Age (years) *	65 (43–83)	67 (50–81)	0.89	66 (43–83)
Gender				
Female	5 (20%)	9 (35%)	0.51	14 (27%)
Male	20 (80%)	17 (55%)		37 (63%)
Site of PT				
Right colon	7 (28%)	11 (42%)		18 (35%)
Left colon	1 (4%)	3 (12%)	0.11	4 (8%)
Rectum	17 (68%)	12 (46%)		29 (57%)
Treatment type				
Chemotherapy + anti-EGFR	19 (76%)	5 (19%)		24 (47%)
Chemotherapy + anti-VEGF	2 (8%)	16 (62%)	<0.001	18 (35%)
Chemotherapy	4 (16%)	5 (19%)		9 (18%)
CEA serum levels *				
≤7.5 ng/mL	10 (40%)	1 (4%)	0.002	11 (22%)
>7.5 ng/mL	15 (60%)	25 (96%)		40 (78%)
Mutational status of PT				
Mutated	16 (64%)	3 (12%)	<0.001	19 (27%)
Wild type	9 (36%)	23 (89%)		32 (63%)
Number of deaths	15 (60%)	21 (81%)	0.009	36 (71%)
Overall survival (months)	52 (38–67)	24 (16–31)	<0.001	40 (30–51)

Results are expressed as the number of cases (percentage) or * as the median (range). PT: primary tumor; CEA: carcinoembryogenic antigen. A primary tumor was considered to be mutated when at least one mutation in the *KRAS*, *NRAS*, *PIK3CA* and/or *BRAF* genes was detected in the patient’s primary tumor.

**Table 2:** Clinical, biological and genetic characteristics of synchronous metastatic colorectal cancer (SMCC) patients (*n* = 51) and their association with overall survival (OS).

Variable	N *	Univariate Analysis	Multivariate Analysis	HR (95% CI)
Age				
<66 years	26 (51%)	0.889		
≥66 years	25 (49%)			
Gender				
Male	37 (76%)	0.879		
Female	14 (24%)			
Site of PT				
Right colon	18 (35%)			
Left colon	4 (8%)	0.114		
Rectum	29 (57%)			
CEA serum levels				
<7.5 ng	11 (22%)	0.073	NS	
≥7.5 ng	40 (78%)			
Treatment type				
Chemotherapy + anti-EGFR	24 (47%)			
Chemotherapy + anti-VEGF	18 (35%)	0.053	NS	
Chemotherapy	9 (18%)			
Microsatellite instability				
No	50 (98%)	0.879		
Yes	1 (2%)			
*KRAS* mutation in PT				
Yes	23 (45%)	0.050	NS	
No	28 (55%)			
*NRAS* mutation in PT				
Yes	1 (2%)	0.487		
No	50 (98%)			
*BRAF* mutation in PT				
Yes	1 (2%)	0.937		
No	50 (98%)			
*PIK3CA* mutation in PT				
Yes	12 (24%)	0.728		
No	39 (76%)			
*KRAS* mutation in plasma				
Yes	23 (45%)	0.004	NS	
No	28 (55%)			
NRAS mutation in plasma				
Yes	3 (6%)	0.420		
No	48 (94%)			
*PIK3CA* mutation in plasma				
Yes	8 (16%)	0.185		
No	43 (84%)			
*BRAF* mutation in plasma				
Yes	2 (4%)	0.472		
No	49 (96%)			
Liquid biopsy at diagnosis				
Positive	26 (53%)	0.005	0.007	0.388 (0.196–0.768)
Negative	25 (47%)			
Mutations in liquid biopsy				
Wild type	25 (49%)	<0.001		
1 mutation	19 (37%)		0.001	2.018 (0.316–3.095)
2 mutations	6 (12%)			
3 mutations	1 (2%)			

* Results are expressed as the number of cases (percentage). PT: primary tumor. A liquid biopsy was considered to be positive when at least one mutation in the *KRAS*, *NRAS*, *PIK3CA* and/or *BRAF* genes was detected in the patient’s plasma. NS: not statistically significant (*p* > 0.05).

**Table 3:** Correlation between the mutational status of the *KRAS*, *NRAS*, *PIK3CA* and *BRAF* genes detected in the plasma and primary tumor of patients (*n* = 51) with synchronous metastatic colorectal cancer (SMCC).

	Plasma											
	*KRAS*		*R/p*	*NRAS*		*R/p*	*BRAF*		*R/p*	*PIK3CA*		*R/p*
Primary Tumor	WT (*n* = 29)	Mutated (*n* = 22)		WT (*n* = 48)	Mutated (*n* = 3)		WT (*n* = 49)	Mutated (*n* = 2)		WT (*n* = 43)	Mutated (*n* = 8)	
*KRAS*												
WT (*n* = 28)	24 (86)	4 (14)	0.65/<0.001									
Mutated (*n* = 23)	5 (22)	18 (78)										
*NRAS*												
WT (*n* = 50)				48 (96)	2 (4)	0.57/0.05						
Mutated (*n* = 1)				0 (0)	1 (100)							
*BRAF*												
WT (*n* = 50)							49 (98)	1 (2)	0.77/<0.001			
Mutated (*n* = 1)							0 (0)	1 (100)				
*PIK3CA*												
WT (*n* = 39)										37 (95)	2 (5)	0.53/0.001
Mutated (*n* = 12)										6 (50)	6 (50)	

Results expressed as number of cases (percentage); WT: wild type; R, correlation coefficient; *p*, probability.

## Data Availability

All study data can be viewed in the manuscript.

## Supplementary Materials

The following supporting information can be downloaded at: https://www.mdpi.com/article/10.3390/ijms24098438/s1.
