# Supplementary material for: Analysis of Circulating Tumor DNA in Synchronous Metastatic Colorectal Cancer at Diagnosis Predicts Overall Patient Survival
Source: Int J Mol Sci. 2023 May 8;24(9):8438. doi: 10.3390/ijms24098438 (PMC10179090; doi:10.3390/ijms24098438)
Supplement: Supplementary file 1 [file ijms-24-08438-s001.zip › ijms-2359777-supplementary.pdf]

Supplementary Table S1. Type of mutations detected in the *KRAS, NRAS, PIK3CA* and *BRAF* genes in paired primary tumor and plasma (liquid biopsy) samples from 51 patients with synchronous metastatic colorectal cancer (SMCC) at diagnosis.

ID	*KRAS*		*PIK3CA*		*NRAS*		*BRAF*	
	PT	Plasma	PT	Plasma	PT	Plasma	PT	Plasma
1	A59T/E/G	A59T/E/G	WT	E545X	WT	WT	WT	WT
2	WT	WT	E542Q	WT	WT	WT	WT	WT
3	Q61H	Q61H	E545X	WT	WT	WT	WT	WT
4	G12V	G12V	H1047X	H1047X	WT	WT	WT	WT
5	WT	WT	E545X	WT	WT	WT	WT	WT
6	WT	WT	E545X	E545X	WT	G12D	WT	WT
7	WT	WT	E545X	E545X	WT	WT	WT	WT
8	G12V	WT	R88Q	WT	WT	WT	WT	WT
9	WT	K117N	E545X	WT	WT	WT	WT	WT
10	WT	WT	WT	E545X	WT	WT	WT	WT
11	WT	G12V	H1047X	E542K	WT	WT	WT	WT
12	G12D	G12D	E545X	E545X	WT	WT	WT	WT
13	G12V	G12V	H1047X	H1047X	WT	WT	WT	V600E
14	WT	WT	E545X	WT	WT	WT	WT	WT
15	WT	WT	WT	WT	WT	WT	WT	WT
16	WT	WT	WT	WT	WT	WT	WT	WT
17	WT	WT	WT	WT	WT	WT	WT	WT
18	WT	WT	WT	WT	WT	WT	WT	WT
19	G12V	WT	WT	WT	WT	WT	WT	WT
20	WT	WT	WT	WT	Q61R/K	Q61R/K	WT	WT
21	G13D	WT	WT	WT	WT	WT	WT	WT
22	G12D	G12D	WT	WT	WT	WT	WT	WT
23	WT	G12D	WT	WT	WT	WT	WT	WT
24	WT	WT	WT	WT	WT	WT	WT	WT
25	WT	WT	WT	WT	WT	WT	WT	WT
26	G13D	G13D	WT	WT	WT	WT	WT	WT
27	WT	WT	WT	WT	WT	WT	V600E	V600E
28	WT	WT	WT	WT	WT	WT	WT	WT
29	WT	WT	WT	WT	WT	WT	WT	WT

30	Q61H	WT	WT	WT	WT	WT	WT	WT
31	WT	WT	WT	WT	WT	WT	WT	WT
32	A146P/T/V	A146P/T/V	WT	WT	WT	WT	WT	WT
33	WT	A59T/E/G	WT	WT	WT	WT	WT	WT
34	G12V	G12V	WT	WT	WT	WT	WT	WT
35	G13D	G13D	WT	WT	WT	WT	WT	WT
36	G13D	G13D	WT	WT	WT	Q61L	WT	WT
37	WT	WT	WT	WT	WT	WT	WT	WT
38	G12V	WT	WT	WT	WT	WT	WT	WT
39	G12A	G12A	WT	WT	WT	WT	WT	WT
40	WT	WT	WT	WT	WT	WT	WT	WT
41	G12V	G12V	WT	WT	WT	WT	WT	WT
42	G12S	G12S	WT	WT	WT	WT	WT	WT
43	WT	WT	WT	WT	WT	WT	WT	WT
44	WT	WT	WT	WT	WT	WT	WT	WT
45	G12D	G12D	WT	WT	WT	WT	WT	WT
46	WT	WT	WT	WT	WT	WT	WT	WT
47	WT	WT	WT	WT	WT	WT	WT	WT
48	G13D	G13D	WT	WT	WT	WT	WT	WT
49	G12A	G12A	WT	WT	WT	WT	WT	WT
50	WT	WT	WT	WT	WT	WT	WT	WT
51	G12C	G12C	WT	WT	WT	WT	WT	WT

PT: Primary tumor; WT: Wild Type
